# The Prevalence of *Cryptosporidium* spp. in Children, Taiz District, Yemen

**Published:** 2010-06

**Authors:** AH Al-Shamiri, AH Al-Zubairy, RF Al-Mamari

**Affiliations:** Department of Applied Microbiology, Faculty of App. Science, Taiz University, Taiz, Yemen

**Keywords:** Prevalence, *Cryptosporidium*, Diagnosis, Children, Yemen

## Abstract

**Background:**

This is the first work done on cryptosporidiosis among the children in Taiz, Yemen.

**Methods:**

A number of 712 samples were collected from children of different ages (ranging from 1 month to 12 years) from Dec 2006 to Aug 2007. The collected samples were examined by Sheather's sugar floatation and Modified Ziehl- Neelsen stain as well as ELISA methods. The test results were statistically analyzed by SPSS software.

**Results:**

The overall positive percentage was 43.7%. The higher incidence (36.2%) was occurred in males while the lowest incidence (32.7%) was observed in females (r=0.876; *P*=0.001). The correlation between infected cases and the type of drinking water was r =0.121. Among the cases examined by ELISA (92 cases), 26.1% were infected. The correlation between seropositivity and gender was r=0.652 (P=0.031).

**Conclusion:**

*Cryptosporidium* spp. is a significant pathogen among children at Taiz. Fresh water supplies, education, eating habits and domestic animals are considered the main sources for transmission of cryptosporidiosis.

## Introduction

*Cryptosporidium* sp. is a pathogen with a worldwide distribution and predicted to be the highest in developing countries especially in children. Diarrhea caused by *C. parvum* in childhood may be associated with subsequent impaired physical and cognitive development (1). Epidemiological studies have indicated that the main routes of transmission of *Cryptosporidium* are human–animal contact, person-to-person and waterborne (2). Numerous reports provide strong evidence that contaminated water is a high risk factor for cryptosporidiosis. In developing countries cryptosporidiosis represent up to 15% of gastrointestinal diseases among children and seroprevalence rates are generally in the 25% to 35% range and often 2 to 3 times higher (3). Those rates suggest that infection can be more common than surveys of fecal oocysts excretion demonstrate, as oocysts may be shed sporadically (4). Some studies on cryptosporidiosis have been conducted in countries such as Iraq (5), Kuwait (6), South Africa (7), Iran (8), and Brazil (9).

Many surveys had been carried out on intestinal parasites in many regions of Yemen. The most frequently found parasites were *Entamoeba histolytica*, *Giardia lamblia*, *Hymenolepis nana*, *Ascaris lumbricoids*, *Ancylostoma duodenale*, *Taenia saginata* and *Schistosoma mansoni* (10, 11). Recently, the epidemiological pattern of cryptosporidiosis and other intestinal parasites were studied among 3 orphanages in Sana'a City (12). In Yemen, local vegetable farmers use manure as fertilizer and obtained drinking water from the water supply systems such as, streams, rivers ponds, and well water (untreated water). Children put so many things into their mouths, and eating habit cause persistent diarrhea.

The aim of this study was to determine the prevalence of *Cryptosporidium* spp. in children diarrheic and non- diarrheic stool and to investigate some risk factors that may be an effect on the prevalence rate of *Cryptosporidium* spp. infection.

## Materials and Methods

### Study area and samples

Taiz Governorate is located about 250 km South of Sana'a, the capital of Yemen, and its climate has many subtropical features. The mean annual temperature is between 20 and 30°C with little seasonal variation. Seven hundred and twelve children stool samples (aged 1 month – 12 years) were collected and examined, 393 of them were among the diarrheic patients attending the out – patient clinic and inpatient section of Yemeni Swedish Hospital MCH (Mother Children Hospital) in Taiz City, Yemen, while 319 samples apparently healthy children (Non diarrheic children). The required data were collected via the redesigned questionnaire. The cases were divided into 3 groups according to the age: the infancy group of age ranging from one month to less than two years, the preschool group, of age varies from 2 years to less than 6 years and the school group with age rang of 6 to 12 years.

### Staining methods and microscopic examination

Fecal samples were collected in clean, labeled waxed cardboard boxes and examined as soon as received by naked eye for consistency. The stool samples were preserved in 10% formalin or frozen under 22°C until used. The samples were concentrated by Sheather's sugar floatation method (13). For detecting the *Cryptosporidium* oocysts, ordinary light microscope with 100 magnification power by oil immersion lens was used. A thin smear of the supernatant was prepared and left to dry for fixation and staining. The smears were fixed in methyl alcohol for 2–3 minutes. The staining method was employed by using Modified Ziehl- Neelsen Stain (14).

### Serological test (ELISA)

For detection of *Cryptosporidium* spp., the DRG ®*Cryptosporidium* Ag (stool) (EIA-3467) (direction for use in vitro diagnosis (DRG international Inc, USA); was used. Ninety-two stool samples were diluted with buffer (1:3 dilutions) and mixed well. After finishing the test according to Kit procedure, the results were read visually at 450/620–650nm (15).

The data were statistically analyzed by SPSS version 11.0.

## Results

Among the 712 stool samples of children examined, 34.7% showed oocysts of *Cryptosporidium* spp. The highest infection by *Cryptosporidium* spp. in different children age groups was observed in preschool group, while the lowest infection was noticed in the infant group. The percentage of infection in diarrheic children (symptomatic cases) of the different age groups was 38.4%. On the other hand, the percentage of infection in non-diarrheic children (asymptomatic cases) of different age groups was 30.1%. The highest positive rate of *Cryptosporidium* spp. in different groups of diarrheic children was noticed in the preschool age group 48.1%, followed by the school age group 43.1%, whereas the lowest positive rate was noticed in infant age group (20.2%). Concerning the non-diarrheic children groups, the highest positive rate of *Cryptosporidium* spp. was occurred in the preschool age group as 31.9% while, the lowest positive rate was 28.1% recorded in the school age group (Table 1). The correlation between diarrheic and non-diarrheic positivity was r*=*0.656. The positive percentage in males was 36.2% and in females was 32.7%. The correlation between gender and positivity was r*=*0.876, *P*=0.001.

**Table 1:** The prevalence rate of *Cryptosporidium* spp. infection in different age groups of diarrheic and non-diarrheic children

		Diarrheic children			Non-diarrheic children			Overall	
		
Age groups	No. of cases	No.	(+)	(%)	No.	(+)	(%)	(+)	(%)
1 month – 2 yr	150	114	23	20.2	36	11	30.6	34	22.7
2 yr – 6 yr	300	156	75	48.1	144	46	31.9	121	40.3
6 yr– 12 yr	262	123	53	43.1	139	39	28.1	92	35.1
Total	712	393	151	38.4	319	96	30.1	247	34.7

Out of the 712 studied cases, 302 were in contact with domestic animals like birds, gooses, and dogs. The higher percentage of positive cases with *Cryptosporidium* spp. (42.4%) was seen in children who were in contact with domestic animals while, the lower percentage (29.1%) was seen in children not in contact with domestic animals. Among the 247 positive cases with *Cryptosporidium* spp., 40.5% used stream water for their drinking followed by (39.6%) who were drinking well water. While a lower percentage (33.1%) was among those who were drinking tank water followed by 25.2% drinking bottled water (Table 2). The correlation between positivity and the type of drinking water was r=0.121 and *P*=0.001. From the 247 positive cases, 43.6% were coming from rural areas, 32.6% from suburban, and 25.1% from urban areas.

**Table 2:** The association between positive cases with *Cryptosporidium* spp. and the type of drinking water

		Positive	
Type of drinking water	No. of Cases	No.	%
Stream water	158	64	40.5
Tank water	163	54	33.1
Bottled water	155	39	25.2
Well water	169	67	39.6
Treated water	67	23	34.3
Overall	712	247	34.7

Out of the cases examined by ELISA method, 26.1% was infected by *Cryptosporidium* spp. The highest percentage (39.2%) seroprevalence was observed among infant group while, the lowest percentage (20.6%) was in preschool age group. However, a higher positive rate (30.7%) was noticed in male group compared to female group (20%). The correlation between seropositivity and the children age groups was r=0.652 (Table 3).

**Table 3:** The positive rate of *Cryptosporidium* spp. of children examined by serological test (ELISA)

Infant group N=28	Preschool group N=63	Total N=92	Male	Female	Total
Positive	Positive	Positive	Positive	Positive	Positive
11 (39.2%)	13 (20.6%)	24 (26.1%)	16 (30.7%)	8 (20%)	24 (26.1%)

## Discussion

Among 712 stool samples examined, 34.7% were positive with Cryptosporidiosis. These results were nearly similar to the results of 3 Yemeni orphanages in Sana'a City (12). They reported the prevalence of *C. parvum* as 24%. Another Egyptian study (16) stated that 44% were positive for this parasite. Cryptosporidiosis is widely spread all over the world with variable prevalence of samples tested ranging from 1.6% in Egypt to 94% in Kuwait (17).

The highest rate of infection by *Cryptosporidium* spp. was 40.3%, which had been observed in preschool age group between 2–6 years old. While the lowest rate of infection was 22.7% which was noticed in infant age group of 1month to 2 years old. These results were in agreement with other studies conducted in Kuwait. The prevalence rate 73% represented children of >2 years compared to 27% of children <2 years of age (6). In Zagazig- Egypt (18), cryptosporidiosis was more common in the age of 2–12 years old. In Korea, the peak incidence of cryptosporidiosis was in children aged 1–5 years (19). Thus, at age of 2 to 6 years of children may be more exposed to the infections by *Cryptosporidium* spp. because they lack the knowledge about the good food and water. They eat without washing their hands, play in soil and sewage water, exposed to more fecal oral contact or through contaminated food or water, or may be attributed to their weak immune responses (8, 20).

The percentage of diarrheic children infected by *Cryptosporidium* spp. was 38.45%, while the percentage in the non-diarrheic children was 30.1%. These results trend to the result of (8) in Iran of which *C. parvum* was detected in 25.6% of diarrheic and 3.7% of non-diarrheic children, but in contrast with Egyptian study with *C. parvum* in 13.9% of children with diarrhea (21).

Regarding gender variation our study showed that the infection was more common among males than females, which could be due to different, sample size of males was higher than females, or may be due to playing of male children in the gardens and farms outdoor area with soil and animals, which can increase the risk of parasite transmission. These results were in accordance another one, where the men were infected at a higher rate (1.9%) than women (1.2%) were (22). The male higher infection rate of *C. parvum* than female was also observed in Guinea-Bissau (23). Another study, reported that the boys and girls had similar detectable positive rate (24).

The contact with domestic animals acts as a risk factor in zoonotic infection (25). A similar result in which the contact with animal reservoirs should be considered (26). There was a high significant association between the cases and type of drinking water. These results were in agreement with a previous study (8). In Korea (22) and in Austria (27) considered the contamination of drinking water as an important factor in the high prevalence of *Cryptosporidium* spp. It has become the most important contaminant found in drinking water and is now considered as high risk water borne disease. In Yemen, the summer season is a rainy season, thus, the heavy rainfall could help the transmission of *Cryptosporidium* spp. by inducing a wider spread of the animal feces onto fields or water sources.

43.6% of cases were coming from rural areas and 25.1% from urban areas and in agreement with previous studies (28, 29) but disagreement with another one (26). This could be due to the social habits of the rural people in which keep the animals in their houses.

The highest percentage (44.7%) was in patients with illiterate parents while the lowest percentage (22.2%) was with educated parents. The correlation between positivity and the level of parents' education was (r*=*0. 804 and *P* value=0.001). This finding revealed that when the level of parents' education increased, the positivity of *Cryptosporidium* spp. infection decreased.

It is found that the seroprevalence in developed countries are generally in the range of 25% to 35% compared to that of developing countries, which is 2–3 times higher (30).

In conclusion*, Cryptosporidium* spp. is a significant pathogen among children in Taiz, Yemen. Fresh water supplies, education, eating habits and domestic animals like cattle; livestock are the main sources for transmission of cryptosporidiosis. Thus, health education for parents to protect their children of being infected with *Cryptosporidium* spp. especially by contaminated water, and hand hygiene following contact with domestic animals must be considered.

## References

[1] Guerrant DI, Moore SR, Lima AA, Patrick PD, Schorling JB, Guerrant RL (1999). Association of early childhood diarrhea and cryptosporidiosis with impaired physical Wetness and cognitive function four-seven years later in a poor urban community in northeast Brazil. Am J Trop Med Hyg.

[2] Meinhart PL, Casemore DP, Miller KB (1996). Epidemiologic Aspects of human cryptosporidiosis and the role of waterborne transmission. Epid Rev.

[3] Juranek DD, Strickland G (2000). Cryptosporidiosis. ThimasHunter's Trop. Med. and Emerging Infectious Disease.

[4] Ungar BL, Gerald L, Mandell J E, Bennett R D (2000). Cryptosporidium. Part (III – infectious diseases and their etiological agents. Principles and practice of infectious diseases.

[5] Mahdi N K, Al-Sadoon IA, Mohamed A T (1996). First report of cryptosporidiosis among Iraqi children. East Mediterr Health J.

[6] Iqbal J, Hira PR, Al-Ali F, Philip R (2001). Cryptospordiosis in Kuwaiti children: Seasonality and endemicity. Clin Microbiol Infect.

[7] Samie A, Bessong P O, Obi CL, Sevilleja JE, Stroup S, Houpt E, Guerrant R L (2006). *Cryptosporidium* species: Preliminary descriptions of the prevalence and genotype distribution among school children and hospital patients in the Venda region, Limpopo province, South Africa. Exper Parasitol.

[8] Mirzaei M (2007). Prevalence of *Cryptosporidium* sp. infection in diarrhea and non-diarrheic humans in Iran. Korean J Parasitol.

[9] Bushen OY, Kohli A, Pinkerton RC, Dupnik K, Newman RD, Sears CL, Fayer R, Lima AA, Guerrant RL (2007). Heavy cryptosporidial infections in children in northeast Brazil: comparison of *Cryptosporidium hominis* and *Cryptosporidium parvum*. Trans R Soc Trop Med Hyg.

[10] Al-Taj MA, Al-Shamiri A H (2004). Prevalence of intestinal parasitic infections among school children in Taiz City, Republic of Yemen. Bull. Fac Sci. Assiut Univ.

[11] Baswaid S H, Al-Haddad AM (2008). Parasitic infections among restaurant workers in Mukalla (Hadhramout/Yemen). Iranian J Parasitol.

[12] Al-Shibani LA, Azazy AA, El-Taweel HA (2009). Crypttosporidiosis and other intestinal Parasites in 3 Yemeni orphanages: prevalence, risk and morbidity. J Egypt Sco Parasitol.

[13] 13. Soave R, Johnson WD (1988). *Cryptosporidium* and *Isospora belli* infections. J Infect Dis.

[14] Henricksen SA, Pohlenz JF (1981). Staining of Cryptosporidia by a modified Ziehl- Neelsen technique. Acta Vet Sacnd.

[15] Goma FY, Geurden T, Siwila J, Phiri IGK, Gabriel S, Claerebout E, Vercruysse J (2007). The Prevalence and Molecular characterization of *Cryptosporidium* spp. in small ruminants in Zambia. Small Ruminant Res.

[16] Helmy MM, Rashed LA, El-garhy MF (2004). Molecular characterization of *Cryptosporidium parvum* isolates obtained from humans. J Egyptian Soc Parasitol.

[17] Sulaiman IM, Hira PR, Zhou L, Al-Ali FM, Al-Shelahi FA, Shweiki HM, Iqbal J, Khalid N, Xiao L (2005). Unique endemicity of cryptosporidiosis in children in Kuwait. J Clin Microbiol.

[18] Abou-El-Magd LA, Abou –Shady O (1986). A preliminary study of human cryptosporidiosis. J Egyptian Soc Parasitol.

[19] Casemore DP (1990). Epidemiological aspects of human Cryptosporidiosis. Epidemiol Infect.

[20] Yu JR, Lee JK, Seo M, Kim SI, Sohn WM, Huh S, Choi HY, Kim TS (2004). Prevalence of cryptosporidiosis among the villagers and domestic animals in several rural areas of Korea. Korean J Parasitol.

[21] Rizk H, Soliman M (2001). Coccidiosis among malnourished children in Mansoura, Dakahlia Governorate. Egypt. J Egypt Soc Parasitol.

[22] Park JH, Kim H.J, Guk SM, Shin EH, Kim JL, Rhim H.J, Lee SH, Chai JY. A (2006). survey of cryptosporidiosis among 2,541 residents of 25 coastal islands in Jeollanam-Do (Province), Republic of Korea. Korean J Parasitol.

[23] Molbak K, Aaby P, Hojlyng N, da Silva AP (1994). Risk factors for *Cryptosporidium* diarrhea in early childhood: a case control study from Guinea-Bissau. West Africa. Am J Epidemiol.

[24] Lu J, Li C, Jiang S, Ye S (2008). The survey of *Cryptosporidium* infect among young children in kindergartens in Anhui province. J Nanjing Med University.

[25] Siwila J, Phiri IG, Vercruysse J, Goma F, Gabriel S, Claerebout E, Geurden T (2007). Asymptomatic cryptosporidiosis in Zambian dairy farm workers and their household members. Trans R Soc Trop Med Hyg.

[26] Yu JR, Lee JK, Seo M, Kim SI, Sohn WM, Huh S, Choi HY, Kim TS (2004). Prevalence of cryptosporidiosis among the villagers and domestic animals in several rural areas of Korea. Korean J Parasitol.

[27] Hassl A, Benyr G, Sommer R (2001). Occurrence of *Cryptosporidium* sp. oocysts in Fecal and water samples in Austria. Acta Trop.

[28] Abdel-Maboud AI, Rossignol JF, Mostafa MS (2000). Cryptosporidiosis in Benha; study of some modalities in diagnosis and treatment. J Egyptian Soc Parasitol.

[29] Chacin-Bonilla L, Bonilla MC, Soto-Torres L, Rios-Candida Y, Sardina M, Enmanuels C, Parra AM, Sanchez-Chavez Y (1997). *Cryptosporidium parvum* in children with diarrhea in Zulia State, Venezuela. Am J Trop Med Hyg.

[30] Hayam ME (2002). Nosocomial sources of cryptosporodial Infect.

